# Sex differences in the expression of calcitonin gene-related peptide receptor components in the spinal trigeminal nucleus

**DOI:** 10.1016/j.ynpai.2019.100031

**Published:** 2019-04-24

**Authors:** Yadong Ji, Alexandra Rizk, Pamela Voulalas, Hanan Aljohani, Simon Akerman, Gregory Dussor, Asaf Keller, Radi Masri

**Affiliations:** a Department of Advanced Oral Sciences and Therapeutics, University of Maryland School of Dentistry, Baltimore, MD, USA; b Program in Neuroscience, University of Maryland School of Medicine, Baltimore, MD, USA; c Department of Anatomy and Neurobiology, University of Maryland School of Medicine, Baltimore, MD, USA; d Department of Oncology and Diagnostic Sciences, University of Maryland School of Dentistry, Baltimore, MD, USA; e Department of Oral Medicine and Diagnostics Sciences, King Saud University, School of Dentistry, Riyadh, Saudi Arabia; f Department of Neural Sciences and Pain, University of Maryland School of Dentistry, Baltimore, MD, USA; g School of Behavioral and Brain Sciences, The University of Texas at Dallas, Richardson, TX, USA

**Keywords:** ACSF, artificial cerebrospinal fluid, CGRP, calcitonin gene-related peptide, CLR, calcitonin receptor-like protein, IM, inflammatory mediators, RAMP1, receptor activity-modifying protein 1, RCP, receptor component protein, shRNA, short hairpin RNA, SpVc, spinal trigeminal nucleus caudalis, Migraine, Headache, Trigeminal, Meninges, Allodynia, CGRP

## Abstract

**Background and purpose:**

Calcitonin gene-related peptide (CGRP) plays an important role in migraine pathophysiology. CGRP acts primarily by activating a receptor composed of 3 proteins: calcitonin receptor-like receptor (CLR), receptor activity-modifying protein 1 (RAMP1), and receptor component protein (RCP). We tested the hypothesis that sex differences exist in protein levels of two key components of this CGRP receptor: CLR and RCP.

**Methods:**

We used specific antibodies to assess baseline protein levels of CLR and RCP in the spinal trigeminal nucleus caudalis (SpVc) and upper cervical spinal cord of both male and female rats. We also tested if manipulations that knock-down the expression of RCP in SpVc, using locally-mediated gene transfer of short hairpin RNA (shRNA), ameliorate pain in an animal model of intracranial migraine-like pain induced by chemical noxious stimulation of the meninges. To assess pain, we used tests of ongoing pain (rat face grimace test and freezing behavior) and tests of facial mechanical hypersensitivity and allodynia.

**Results:**

There was no difference in CLR levels between male and female animals (p > 0.11) in SpVc and the upper cervical cord. However, female animals exhibited greater baseline levels of RCP (up to 3-fold higher) compared to males (p < 0.002). The knock-down of RCP expression in SpVc attenuated mechanical facial allodynia induced by chemical noxious stimulation of the meninges, but had little effect on ongoing pain behaviors in female and male animals.

**Conclusions:**

RCP is an integral component of the CGRP receptor and may play a key role in mediating CGRP induced central sensitization after noxious stimulation of the meninges. RCP expression in the SpVc and upper cervical cord is sexually dimorphic, with higher levels of expression in females. This dimorphism may be related to the increased incidence of migraines in females–a hypothesis that should be tested in the future.

## Highlights

• Protein expression of receptor component protein (RCP)—a part of the calcitonin gene related peptide receptor (CGRP)—in the spinal trigeminal subnucleus caudalis (SpVc) and upper cervical cord is sexually dimorphic, with higher baseline levels of expression in female than male rats.
• The knock-down of RCP expression the SpVc attenuates mechanical facial allodynia induced by chemical noxious stimulation of the meninges, but have little effect on ongoing pain behaviors in female and male animals.
• CGRP receptor components can be potential therapeutic targets for the treatment of migraine headache.

## 1 Introduction

Migraine afflicts hundreds of millions of individuals worldwide, with a pronounced sex difference as migraine is 3 times more prevalent in females than males (Lipton and Stewart, 1998). Migraine-related symptoms and migraine-related disability are also more frequent in females (Buse et al., 2013, Chai et al., 2014, Pavlovic et al., 2017, Agosti, 2018). The cause for sex-related difference in migraine is multifactorial. Sex hormones are likely a major contributor, but there are other anatomical, biological and psychosocial factors that may contribute to this difference (Bolay et al., 2011, Bolay et al., 2015, Chai et al., 2014, Pavlovic et al., 2017).

Accumulating evidence suggests that calcitonin gene-related peptide (CGRP) plays a key role in migraine pathophysiology (Iyengar et al., 2017, Labastida-Ramírez et al., 2017). CGRP is released when meninges and meningeal afferents are stimulated (Hanko et al., 1985, Goadsby et al., 1988, Zagami et al., 1990, Cathcart et al., 2010), and intravenous infusion of CGRP can induce migraine-like attacks in migraineurs (Lassen et al., 2002, Lassen et al., 2008, Durham, 2004, Rudolf et al., 2005, Hansen et al., 2010, Deen et al., 2017). In addition, plasma, and saliva levels of CGRP in humans are significantly elevated during a migraine attack (Goadsby et al., 1988, Goadsby and Edvinsson, 1993, Jang et al., 2011), and CGRP is elevated in chronic migraineurs during a migraine attack (Cernuda-Morollón et al., 2013, Riesco et al., 2017). Triptans (anti-migraine agents) can normalize CGRP levels, with a concurrent resolution of migraine (Gallai et al., 1995, Sarchielli et al., 2000), and small molecule CGRP antagonists and antibodies are a promising drug class for episodic and chronic migraine treatment (Negro et al., 2012, Goadsby et al., 2017, Dodick et al., 2018).

CGRP is widely distributed in the trigeminovascular system (Lennerz et al., 2008, Eftekhari and Edvinsson, 2011, Bhatt et al., 2014, Eftekhari et al., 2015, Miller et al., 2016) including the spinal trigeminal nucleus caudalis (SpVc)–a nucleus heavily implicated in central mechanisms of migraine (Moskowitz, 1990, Goadsby et al., 1991, Kaube et al., 1993, Cutrer et al., 1995, Burstein et al., 2004, Burstein and Jakubowski, 2010, Iyengar et al., 2017, Lukács et al., 2017). Differences in CGRP expression and distribution may contribute to the sex-related differences in migraine. In female rats, the basal levels of CGRP-encoding mRNA are high in areas containing the SpVc, compared to males (Stucky et al., 2011), and the application of CGRP to the meninges produces cutaneous facial and hindpaw allodynia only in female, but not in male rats (Burgos Vega et al., 2018).

CGRP acts by activating a receptor that is a heterodimer of two membrane proteins: calcitonin receptor-like receptor (CLR) and receptor activity-modifying protein 1 (RAMP1) (McLatchie et al., 1998, Dickerson, 2013, Edvinsson and Warfvinge, 2013). CLR is a member of the class B/Secretin family of G protein-coupled receptors (GPCRs) that are activated by an array of neuropeptides, peptide endocrine hormones, and peptide paracrine factors (Hoare, 2005). The RAMP1 transmembrane protein confers ligand specificity of the CGRP receptor to CGRP (McLatchie et al., 1998, Hong et al., 2012, Hay and Pioszak, 2016). In addition to defining ligand specificity, physical association of CLR with RAMP is required for translocation of the functional heterodimer to the cell surface (McLatchie et al., 1998). A third CGRP receptor component, receptor component protein (RCP), a low molecular weight, hydrophilic, membrane-associated protein, is required to form a functional CGRP receptor (Luebke et al., 1996, Evans et al., 2000, Prado et al., 2001). RCP expression is increased during inflammatory pain (Ma et al., 2003). Its expression correlates with CGRP efficacy and enhances receptor function in response to CGRP (Naghashpour et al., 1997, Goharkhay et al., 2007).

SpVc of female rats has lower levels of all three CGRP receptor components mRNA encoding genes (CLR, RAMP1, and RCP) than males (Stucky et al., 2011). However, it is not known if lower mRNA levels of CGRP receptor components translates to lower levels of protein expression. Here, we compare protein levels of key components of the CGRP receptor in male and female animals. We test the null hypothesis that there is no difference in protein expression of CGRP receptor components in the SpVc and upper cervical spinal cord between male and female rats.

## 2 Materials and methods

We adhered to accepted standards for rigorous study design and reporting to maximize the reproducibility and translational potential of our findings as described in (Landis et al., 2012) and in ARRIVE (Animal Research: Reporting In Vivo Experiments) guidelines. The animals were randomly allocated to respective groups tested, as described in (Kim and Shin, 2014). In all behavioral experiments the investigators were blinded to animal condition. A coded key of all specimens evaluated was kept and not shared with the investigators performing the experiments until data analyses were completed. We performed a power analysis to estimate the required samples needed for each experiment.

### 2.1 Animals

Sprague-Dawley rats of both sexes (M: n = 29, F: n = 30, 9–12-weeks old) weighing 250–300 g (Envigo, Frederick, MD) were used. All animals were housed in climate-controlled facilities with a 12 h light/dark cycle and free access to food and water. Experiments were performed during the light cycle. Protocols for the care and use of the experimental animals were approved by the Institutional Animal Care and Use Committee, and conformed to the guidelines of the International Association for the Study of Pain and the principles set forth in the National Institutes of Health Guide for the Care and Use of Laboratory Animals.

### 2.2 Tissue collection and preparation of protein extracts

Animals were euthanized and the brain was removed with the medulla and brainstem attached. The medulla and brainstem were dissected from the brain such that approximately 1 mm of cerebellar tissue remained attached at the anterior end, with 8 mm of medulla and brainstem remaining at the posterior end. This tissue, which included the SpVc and upper cervical region (C1, C2), was frozen in isopentane on dry ice at −50 °C for 1 min and stored at −80 °C pending further processing. The time between decapitation and snap freezing of tissue was kept to <1 min. Prior to preparation of tissue extracts, the frozen tissues were placed into a −20 °C cryostat for one hour. The excess cerebellar tissue was then removed and the brainstem trimmed coronally at the level of the Obex such that 6.5 mm of SpVc and upper cervical spinal cord-containing tissue remained. We focused on these tissues because they are heavily involved in the relay of headache-related somatosensory information (Classey et al., 2001). This tissue was bisected at the midline into the right and left halves, which were stored separately at −80 °C until the time that tissue extracts were prepared.

The approach for tissue processing was based on our previously published method (Voulalas et al., 2011). Tissue samples were homogenized in ice-cold buffer comprised of 10 mM HEPES pH 7.4, 0.32 M sucrose, 2 mM EDTA, Complete Mini protease inhibitor cocktail (Roche Applied Science, Indianapolis, IN), and phosphatase inhibitors (5 mM NaF, 1 mM NaVO4) using 18–20 S in a Potter-Elvehjem homongenizer. The homogenate was first centrifuged at 1000×*g* for 10 min to pellet nuclei and cellular debris, and the supernatant was then centrifuged at 100,000×*g* for 45 min at 4 °C to sediment cellular membranes. The supernatant was collected and reserved as a crude cytosolic fraction. The crude membrane pellets were resuspended in a buffer comprised of 25 mM HEPES pH 7.4, 2 mM EDTA, Complete Mini protease inhibitors, and phosphatase inhibitors (see above). Crude membranes were further separated into detergent resistant and detergent soluble membrane fractions by addition of Triton X-100 (final concentration 1% v/v) overnight at 4 °C, followed by centrifugation at 100,000×*g* at 4 °C for 45 min. Protein concentration was determined using the Pierce BCA protein assay kit (Thermo Fisher, Waltham, MA).

### 2.3 Western blot analysis

Equal amounts of protein from either the cytosolic, detergent soluble and detergent-resistant membrane fractions were loaded and separated on NuPage® 4–12% Bis-Tris gels with MES running buffer (Invitrogen, Carlsbad, CA). Gels were transferred to nitrocellulose membranes at 30 V for 16 h at 4 °C. Membranes were stained post-transfer with 2% Ponceau S to confirm even lane loading, then blocked with 10% dry milk/10 mM Tris, 150 mM NaCl, 1% Tween 20 (TBST) and probed with primary antibody in 5% milk/TBST. Primary antibodies used in this study included: RCP (rabbit polyclonal #139264, Abcam, Cambridge, MA or mouse monoclonal sc-343347, Santa Cruz Biotechnology, Dallas, TX – both yielded identical results), CLR (rabbit polyclonal sc-30028, Santa Cruz Biotechnology, β-actin (mouse monoclonal A-5316, Sigma-Aldrich, St. Louis, MO), β3-tubulin (T-8660, Sigma-Aldrich). RAMP1 protein was undetectable; despite attempting western blotting with a number of commercially available primary antibodies and therefore we focused our analysis on detecting CLR and RCP. Secondary antibodies used were horseradish peroxidase (HRP)-conjugated goat anti-rabbit (7074, Cell Signaling Technology) and goat anti-mouse (04-18-15, KPL, Gaithersburg, MD). Pierce West Pico and West Femto chemiluminescent substrates were used to visualize immunoreactivity. Chemiluminescent images were obtained with Fuji Super RX-N film, and subsequently scanned and quantified by densitometry using Image Studio Lite (v. 3.1, LI-COR Biosciences, Lincoln, NE).

### 2.4 Survival surgery

*Cannula guide implantation:* Strict aseptic surgical procedures were used in accordance with the University of Maryland’s Guidelines. A presurgical assessment, including weight, behavior, and signs of disease, was conducted for each animal and recorded along with a detailed record of the surgical and post-surgical procedures.

Animals were anesthetized with isoflurane inhalant (3–5% initiation, 1.5–2.5% maintenance). Surgical sites were prepared by removing the hair with an electric clipper and wiped with 10% Betadine surgical scrub then 70% alcohol. An ocular protective lubricant was applied to the animal’s eyes. The animal was then attached to a stereotaxic frame and placed on a thermo-regulated heating pad. Depth of anesthesia was determined every 15 min by monitoring pinch withdrawal, eyelid reflex, corneal reflex, respiratory rate, and vibrissae movements. A long acting local anesthetic (0.5% Marcaine) was applied to the surgical area to further reduce the possibility that animals could experience pain.

A longitudinal incision (5 mm) was performed using a #15 scalpel blade to expose the cranium. The skin and underlying periosteum were reflected using a periosteal elevator. An osteotomy (1 mm diameter) in the skull overlying the right transverse sinus (6.5 mm posterior and 3.0 mm lateral to bregma) was made using a manual drill (DH-0 Pin Vise; Small Parts Inc.). A guide cannula 23 G (O.D.: 0.64 mm; I.D.: 0.32 mm) was loaded with a dummy probe to prevent obstruction of the guide cannula and inserted with caution into the opening, avoiding penetration of the meninges (depth, 1 mm). Two metal screws (TX00-2-C, Small Parts Inc.) were inserted proximal to the osteotomy site and the cannula guide was stabilized with the aid of dental resin.

*Virus injections:* In a subset of animals (females, n = 12, males, n = 12), we injected a replication-deficient lentivirus vector containing 3 target-specific constructs that encode 19–25 nucleotides of shRNA designed to knock-down RCP gene expression (Santa Cruz Biotechnology). Lentiviral vectors reach maximal expression 2–5 days post-injection, and stably integrate into the host genome (Naldini et al., 1996a, Naldini et al., 1996b, Van den Haute et al., 2003, Dittgen et al., 2004), allowing us to achieve a persistent knock-down of RCP expression. In control experiments, a lentivirus with a scrambled sequence of the shRNA was injected.

Virus injections were performed at the time of cannula implantation survival surgery. A laminectomy to expose SpVc was performed. A Hamilton syringe (25 GA) loaded with the recombinant virus was advanced slowly to the target area (from Obex: 3 mm caudal, 2 mm lateral, 0.6 mm depth) and the virus (1 μL) was pressure injected (ipsilateral to the cannula) slowly over a period of 10 min. We targeted the SpVc because in pilot experiments inflammatory mediator (IM) administration to the meninges induced significant *c-Fos* expression in this area.

At the end of survival surgery, the skin was closed with monofilament sutures (4–0, Vicryl). Animals recovered from anesthesia on a thermo-regulated pad, where they were observed every 15 min before being returned to their home cage. Rimadyl 5 mg/kg SQ was given before surgery and SID every 24 h for 48 h. The, rats were allowed to recover for at least one week before commencing with behavioral testing commenced.

### 2.5 Chemical noxious stimulation of the meninges

We used a mixture of IMs for chemical noxious stimulation of the meninges. The IM solution contained 1 mM each of histamine, serotonin, bradykinin, and 0.1 mM prostaglandin E2 in phosphate-buffered saline (PBS) pH 7.4 (Strassman et al., 1996, Oshinsky and Gomonchareonsiri, 2007). In some experiments, we used acidic artificial cerebrospinal fluid (acidic ACSF: 119 mM NaCl, 2.5 mM KCl, 2.5 mM CaCl2, 1 mM NaH2PO4, 26 mM Na2HCO3, 11 mM glucose, pH 5.5) to stimulate the meninges. To administer IM or acidic ACSF, the animals were restrained briefly using a custom made black sock. The dummy cannula was removed and cannula was connected to a polyethylene tube (PE50), and infusion was driven by a microinfusion pump (WPI Inc., Sarasota, FL, USA). The pump allowed for the infusion of 10 μL of IM or acidic ACSF over five minutes. Following the administration of IM or acidic ACSF, the cannula was removed and the dummy cannula was reinserted. The skin surrounding the cannula was visually inspected to insure IM/acidic ACSF did not contact the skin, potentially causing skin irritation. Rats were then removed from the restraining sock and behavioral testing commenced. IM or acidic ACSF were applied only once per animal. All cannula placements on top of the dura were verified post mortem.

### 2.6 Behavioral testing

Animals were handled and acclimatized to the experimenter before and after survival surgery. Handling and acclimatization involved 3 sessions/week whereby animals were gently held, habituated to restraint, and stroked around the periorbital area. Periorbital mechanical withdrawal thresholds were assessed before surgery and after surgery to ensure that thresholds returned to their baseline values before behavioral testing. Behavioral testing started one week after survival surgery and involved assessing animals’ facial grimace (RGS), freezing behavior, and facial mechanical withdrawal thresholds. Testing was performed before (baseline) and after the administration of noxious chemical stimulation to the dura on two separate days, one week apart.

*Rat face grimace score (RGS):* Animals were placed in a Plexiglas chamber (8″ × 8″ inches), and video camera images were recorded for 30 min. To score facial expressions we used the “face finder” application (Sotocinal et al., 2011)—generously provided by Mogil—to capture appropriate screen shots for scoring. We assessed four “action units”: orbital tightening, nose-cheek bulge, vibrissa changes and ear position. Face images were screened, labeled, randomly scrambled and scored, with the experimenter blinded to the treatment groups (baseline vs IM, or baseline vs acidic ACSF) and identity of each image. Ten screenshots were selected for each animal—per group—and on each image, each action unit was given a score of 0, 1, or 2, as previously described (Langford et al., 2010, Sotocinal et al., 2011, Akintola et al., 2017). Mean grimace scores were calculated as the average score across all the action units.

*Freezing behavior:* Video recordings used to determine grimace scores were also used to assess freezing behavior. An investigator blinded to the experimental manipulation examined the recordings and documented the time spent freezing for each animal over a period of 30 min at baseline, and after IM or acidic ACSF application. Freezing was defined as epochs of immobility characterized by increased frequency of breathing and absence of any movements including the vibrissa for periods that lasted more than 10 s/epoch. The time spent freezing was calculated for each animal and averaged across animal in each group.

*Mechanical withdrawal thresholds:* A series of calibrated von Frey filaments were applied to the periorbital area of the face. An active withdrawal of the head from the probing filament was defined as a response. We used the up-down method to determine withdrawal thresholds, as described previously (Chaplan et al., 1994). Responses to von Frey filaments were tested before the application of IM, or acidic ACSF (baseline), and at 30 min, and 60 min after.

### 2.7 Data analysis

We compared the levels of each protein, in each membrane fraction between male and female animals using student *t*-test. Two-way Analysis of Variance was used to analyze RGS and freezing behavior data. The two dependent factors were sex (male vs female) and condition (baseline vs IM/acidic ACSF). Dunnett’s multiple comparison test was used for post-hoc analysis. The Kruskal-Wallis test was used to analyze changes in mechanical withdrawal thresholds, followed by Mann-Whitney *U* test to identify difference between the groups. In all experiments, a p ≤ 0.05 was considered significant. Error bars = 1 standard deviation.

## 3 Results

### 3.1 RCP levels are higher in female animals

**Fig. 1:**
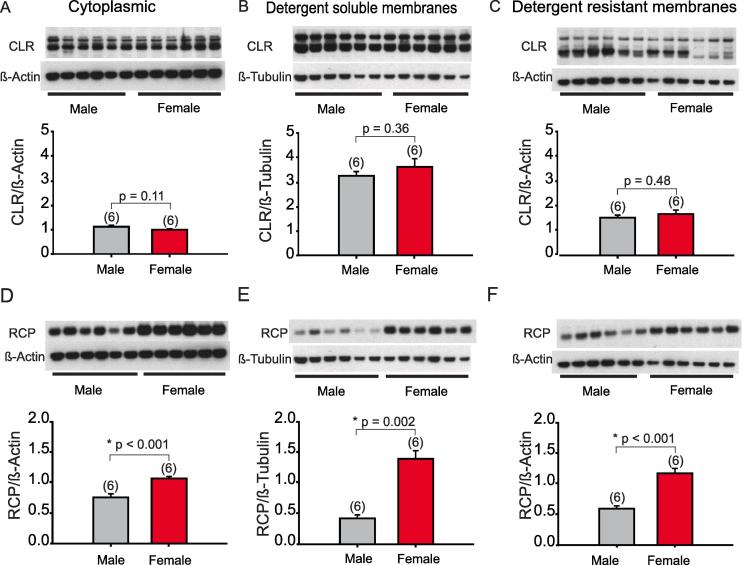
Distribution of CLR and RCP in the SpVc and upper cervical spinal cord. (A) Western blots of CLR relative to β-actin, or β-tubulin in female (n = 6) and male (n = 6) rats (control) in cytoplasmic, (B) detergent soluble or (C) detergent resistant fractions of the homogenized tissues. Quantitation of protein levels is shown below each blot. In E–F, the same animals were used to assess changes in the RCP levels in the same fractions. Here and in all figures, error bars = Standard deviations (SD).

We used western blot analysis to study the expression of two components of the CGRP receptor in SpVc and upper cervical spinal cord homogenates of adult male (n = 6) and female (n = 6) rats (see Methods). In Fig. 1A–C, the expression of CLR in different cellular fractions is shown. There was no significant difference in CLR levels between males and females in the cytoplasmic fraction (p = 0.11, *t*-test), detergent soluble fraction (p = 0.36, *t*-test), or the detergent resistant fraction (p = 0.48).

The expression of RCP, however, was significantly different between males and females (Fig. 1D–F). Cytoplasmic levels of RCP in SpVc and upper cervical cord homogenates were significantly higher in females than in males (p < 0.001, *t*-test). Females also demonstrated a significantly higher level of RCP in detergent soluble membranes (p = 0.002, *t*-test) as well as a higher expression of RCP in detergent resistant membranes (p < 0.001, *t*-test). To our knowledge, this is the first demonstration that RCP protein levels are higher in the SpVc and upper cervical cord of female animals, compared to males.

### 3.2 Meningeal application of IM causes pain

**Fig. 2:**
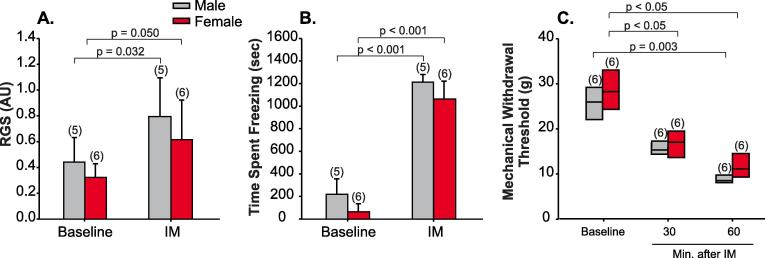
IM application causes ongoing pain and facial allodynia. (A) Rat face grimace scores (RGS) assessed at baseline (1 week after cannula implantation surgery) and immediately after a single application of IM in male (n = 5) and female (n = 6) animals (means ± SD). (B) Freezing behavior determined in the same animals presented in A ((means ± SD). (C) Whisker plot of peri-orbital mechanical withdrawal thresholds assess at baseline (1 week after cannula implantation) and 30 and 60 min after the application of IM in male (n = 6) and female (n = 6) animals.

We asked if RCP levels influence responses to noxious meningeal stimulation in an animal model of intracranial migraine-like pain. We implanted a cannula above the meninges to apply IM or acidic ACSF, to stimulate meningeal afferents. We assessed changes in rat facial grimace scores (RGS), freezing behavior, and mechanical allodynia (see Methods). RGS was significantly increased in both males (F = 9.8, p = 0.03, 2-Way ANOVA, n = 5) and females (p = 0.05, n = 6) after the application of IM (Fig. 2A). There was no difference in RGS between males and females either before (F = 2.1, p = 0.4, 2-Way ANOVA) or after (p = 0.2) IM application. Meningeal application of IM also resulted in increased freezing behavior (see Methods) of both male (F = 399.6, p < 0.001, 2-Way ANOVA, n = 5) and female animals (p < 0.001, n = 6) after the application of IM (Fig. 2B). These findings suggest that IM application results in ongoing pain.

IM application also resulted in a significant reduction of facial mechanical withdrawal thresholds in both male (n = 6) and female (n = 6) animals. In male animals, withdrawal thresholds became significantly lower than baseline values 60 min after IM application (p = 0.003, Kruskal-Wallis). In females, withdrawal thresholds were significantly lower than baseline at both 30 and 60 min after IM application (p < 0.05, Fig. 2C). These findings indicate that both male and female animals develop mechanical allodynia after IM application, and are consistent with previous reports on the effect of meningeal IM applications on male (Edelmayer et al., 2009, Edelmayer et al., 2012) and female rats’ behavior (Stucky et al., 2011).

### 3.3 RCP knock-down ameliorates allodynia

**Fig. 3:**
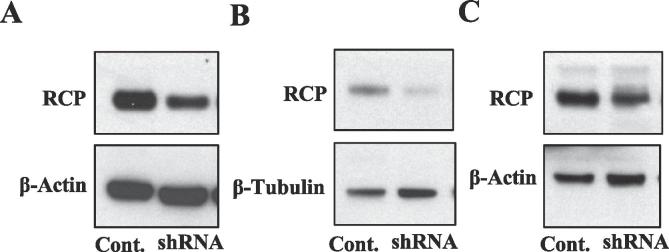
RCP shRNA knocks-down RCP protein levels. Examples of western blot of homogenized brain tissues treated with a Lenti virus to express RCP shRNA. In control experiments scrambled RCP shRNA was expressed. RCP shRNA knocked-down RCP expression in all fractions tested: (A) Cytoplasmic, (B) Detergent soluble, and (C) Detergent resistant fractions.

To test if RCP levels contribute to ongoing pain and allodynia after IM application, we injected a viral vector that causes the expression of RCP shRNA to locally knock-down levels of RCP in the SpVc (see Methods). Fig. 3 shows an example of RCP expression in brain tissues treated with RCP shRNA compared to controls (scrambled RCP shRNA sequence), demonstrating that RCP shRNA results in the knock-down of RCP levels in all fractions investigated. RCP expression in SpVc of animals treated with RCP shRNA was significantly lower (RCP/β-actin: 63.8 ± 16.7%, n = 6, p = 0.02, *t*-test) than in animals injected with the control virus (92.6 ± 8.1%, n = 6).

**Fig. 4:**
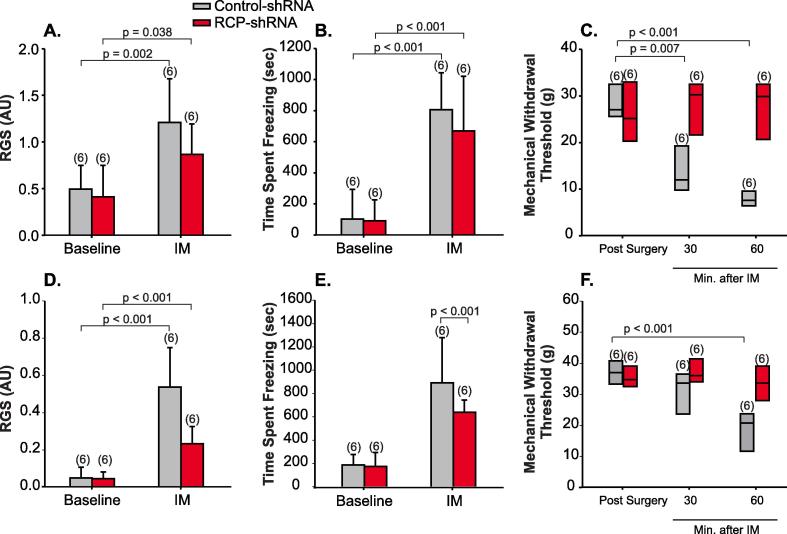
RCP shRNA blocks IM-induced allodynia but not ongoing pain. (A) RCP shRNA (n = 6) or control shRNA (n = 6) had no effect on RGS scores or (B) freezing behavior at baseline, or after the application of IM of female rats. Control-shRNA virus expressed a scrambled sequence of RCP shRNA. (C) Whisker plots of mechanical withdrawal thresholds induced by IM application in animals treated with RCP shRNA in SpVc (n = 6) and animals treated with control shRNA (n = 6). RCP shRNA blocked IM-induced facial allodynia. In male animals, (D) face grimace scores and (E) freezing behavior increased significantly after the injection of IM, even in animals treated with RCP shRNA. (F) Whisker plots of mechanical withdrawal thresholds induced by IM application in male animals treated with RCP shRNA in SpVc (n = 6) and animals treated with control shRNA (n = 6). As in females, RCP shRNA blocked IM-induced facial allodynia.

RCP knock-down had no effect on behaviors that assess ongoing pain induced by IM application: RGS and freezing behavior. After IM application, RGS increased significantly in female animals where RCP was knocked-down (p = 0.04, 2-Way ANOVA, n = 6) and in control animals (p = 0.002, n = 6, Fig. 4A). IM application also resulted in increased time spent freezing in the two groups (RCP knock-down: p < 0.001, control: P < 0.001, 2-Way ANOVA, n = 6/group, Fig. 4B). We observed similar results in male animals (Fig. 4D and E).

Mechanical withdrawal thresholds were significantly reduced in control female animals 30 (p = 0.007, Kruskal-Wallis, n = 6) and 60 min (p < 0.001) after IM application, but not in RCP-knockdown animals (p = 0.498, Fig. 4C). Similar results were also observed for male animals (Fig. 4F). These results suggest that RCP knock-down in SpVc blocks IM-induced allodynia, but not ongoing pain.

**Fig. 5:**
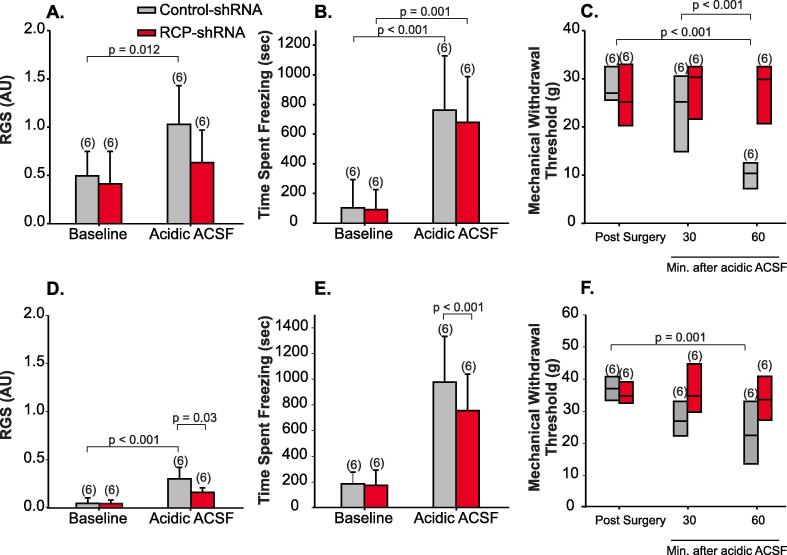
RCP shRNA blocks acidic ACSF-induced allodynia but not ongoing pain. (A) RCP shRNA (n = 6) reduced RGS scores but not (B) freezing behavior of female rats receiving applications of acidic ACSF to the meninges. (C) Whisker plots of mechanical withdrawal thresholds induced by acidic ACSF application in animals treated with RCP shRNA in SpVc (n = 6) and animals treated with control shRNA (n = 6). RCP shRNA blocked acidic ACSF-induced facial allodynia. In male animals, (D) face grimace scores and (E) freezing behavior increased significantly after the injection of ACSF, even in animals treated with RCP shRNA. (F) Whisker plots of mechanical withdrawal thresholds induced by ACSF application in male animals treated with RCP shRNA in SpVc (n = 6) and animals treated with control shRNA (n = 6). As in females, RCP shRNA blocked IM-induced facial allodynia.

We also tested the effects of RCP knock-down on behavioral responses to the administration of another noxious chemical: acidic ACSF (see Methods). Acidic ACSF activates distinct ion channels in the meninges, acid sensing ion channels, and induces pain behaviors in rats (Yan et al., 2011, Yan et al., 2013). In both control and RCP knock-down animals, RGS was increased after the application of acidic ACSF in female animals, however, this difference was only significant in control animals (p = 0.012, 2-Way ANOVA, n = 6/group, Fig. 5A). Both groups demonstrated a significant increase in time spent freezing after the injection of acidic ACSF in female animals (control: p < 0.001, ACSF: p = 0.001, Fig. 5B). Facial mechanical withdrawal thresholds decreased for the control group 60 min after the injection of ACSF (p < 0.001, Kruskal-Wallis), but RCP knock-down blocked the development of allodynia (p = 0.132, Fig. 5C). The injection of ACSF in male animals produced similar behavioral effects to those observed in females and RCP knock-down in SpVc appeared to primarily block the development of mechanical allodynia (Fig. 5D-E) than ongoing pain behaviors after noxious stimulation of meningeal afferents.

## 4 Discussion

We tested if there are sex differences in the expression of CLR and RCP—two key components of the CGRP receptor—in the brainstem containing SpVC. Our null hypothesis was that there is no difference in protein expression of CGRP receptor components in the SpVc between male and female rats. Our findings partially reject the null hypothesis. While there were no baseline sex differences in protein levels of CLR, we discovered that the expression of RCP was significantly greater (up to 3-fold increase) in female than in male rats. We also discovered that knock-down of RCP levels ameliorates mechanical allodynia in an animal model of migraine headaches, but had little effect on ongoing pain behaviors. These findings suggest that RCP may play a role in CGRP signaling in the SpVc following intracranial meningeal stimulation and may contribute to sex differences observed in migraine.

RCP expression is increased during inflammatory and neuropathic pain and is thought to increase CGRP receptor sensitivity to CGRP (see Introduction). When trigeminovascular afferents are activated, CGRP is released, along with other inflammatory mediators that contribute to vasodilation, neurogenic inflammation and nociceptive transmission (Iyengar et al., 2017). In pathologic conditions, the release of CGRP and subsequent activation of CGRP receptors modulate RCP expression (Ma et al., 2003). Increased RCP levels at baseline may enhance the efficacy of CGRP-dependent responses to stimuli and may contribute to an exacerbated pain response. It is important to note however that RCP is not specific for CGRP receptors but is also part of the adrenomedullin receptor that may also contribute to pain mechanism in the trigeminal complex (Walker and Hay, 2013). Additional experiments are required to further understand how increased levels of RCP influence specific receptor function within the SpVc.

A previous study demonstrated that, in the spinal trigeminal complex, mRNAs encoding the CGRP receptor components—RAMP1, CLR, and RCP—are lower in female rats, compared to male (Stucky et al., 2011). Thus, we predicted that protein levels of these components would also be lower in female rats. However, we found that RCP protein levels are *higher* in females, while CLR levels did not vary between sexes. Differences in processes that regulate transcription and translation may explain this dichotomy (see: (Pereira et al., 2005). In some instances, the presence of RNA binding proteins that upregulate translation may produce elevated protein levels even when transcription is low. Additionally, post-translational modifications, such as phosphorylation, acetylation, or glycosylation may increase the stability of the protein, resulting in an apparent increase in protein levels even if encoding mRNA levels are low (Vélez-Bermúdez and Schmidt, 2014). Additional work is necessary to identify how the regulation of transcription and translation of CGRP receptor components differ between male and female animals.

Consistent with the literature, meningeal application of IM or acidic ACSF induced characteristic behaviors that suggest the presence of ongoing pain and facial, periorbital allodynia (Edelmayer et al., 2009, Edelmayer et al., 2012, Stucky et al., 2011, Yan et al., 2011, Yan et al., 2013, Alabwah et al., 2016). In females, IM-induced mechanical allodynia appeared earlier than in male animals (within 30 and 60 min after application versus at least 60 min following application in males). These findings are consistent with a previous report demonstrating lower IM doses are needed to induce pain behaviors in female rats and that pain behaviors last longer in female animals compared to males (Stucky et al., 2011).

RCP knock-down in the SpVc blocked the development of periorbital allodynia but had little effect on RGS and freezing behavior in female rats. Animal studies suggest that the development of allodynia in migraine is due to the development of central sensitization (Burstein et al., 2004, Burstein and Jakubowski, 2010). As such, our data support previous findings that RCP is an integral component of the CGRP receptor, and suggest that CGRP transmission, in SpVc, may play a key role in mediating central sensitization after IM/acidic ACSF application (Fischer et al., 2005, Sixt et al., 2009, Fischer et al., 2018).

In addition, we compared the effect of IM and acidic saline to that of animal responses before the administration of algesic substances and did not use vehicle injections as a control. We reasoned that the administration of control fluids onto the meninges is not an appropriate control because the injection is also likely to mechanically sensitize the meninges as has been reported in the literature (e.g.: increased calcitonin gene related peptide expression due to control injections onto the dura (Stucky et al. 2011)).

Following the recommendation of the International Association for the Study of Pain, we assessed the expression of CGRP receptor component protein in gonadally intact male and female rats as a first step to determine if sex differences exist (Greenspan et al., 2007). However, future studies in cycling females, ovariectomized females and males and females with hormonal manipulations will be necessary to further determine the effect of gonadal hormones on RCP receptor components.

In summary, RCP—a key component of the CGRP receptor—is expressed at higher levels in the SpVc and upper spinal cord of female animals compared to males. The CGRP receptor represents an essential regulatory point for CGRP signaling, and the baseline differences in RCP expression may contribute to sex-related differences in migraine. RCP may be a strong candidate for pharmacologic intervention to ameliorate signs of pain.

## Funding Acknowledgement

Research reported in this publication was supported by the National Institute of Neurological Disorders and Stroke of the National Institutes of Health grants R01NS099245 (AK & RM) and R01NS104200 (GD). The content is solely the responsibility of the authors and does not necessarily represent the official views of the National Institutes of Health. The funding sources has no role in study design; the collection, analysis and interpretation of data; the writing of the report; or in the decision to submit the article for publication.

## Conflict of interest

The authors declare no conflict of interest.
